# Mid-Infrared Imaging Is Able to Characterize and Separate Cancer Cell Lines

**DOI:** 10.1007/s12253-020-00825-z

**Published:** 2020-06-16

**Authors:** E. Kontsek, A. Pesti, M. Björnstedt, T. Üveges, E. Szabó, T. Garay, P. Gordon, S. Gergely, A. Kiss

**Affiliations:** 1 2nd Department of Pathology, Semmelweis University, Budapest, Hungary; 2 Laboratory for Clinical Pathology and Cytology, Department of Laboratory Medicine, Division of Pathology, Karolinska Institutet, Karolinska University Hospital, Stockholm, Sweden; 3 Department of Applied Biotechnology and Food Science, Budapest University of Technology and Economics, Budapest, Hungary; 4 Department of Electronics Technology, Budapest University of Technology and Economics, Budapest, Hungary

**Keywords:** Cancer, FT-IR, Fingerprint region, Transflectance

## Abstract

Malignancies are still responsible for a large share of lethalities. Macroscopical evaluation of the surgical resection margins is uncertain. Big data based imaging approaches have emerged in the recent decade (mass spectrometry, two-photon microscopy, infrared and Raman spectroscopy). Indocianine green labelled MS is the most common approach, however, label free mid-infrared imaging is more promising for future practical application. We aimed to identify and separate different transformed (A-375, HT-29) and non-transformed (CCD986SK) cell lines by a label-free infrared spectroscopy method. Our approach applied a novel set-up for label-free mid-infrared range classification method. Transflection spectroscopy was used on aluminium coated glass slides. Both whole range spectra (4000–648 cm^−1^) and hypersensitive fingerprint regions (1800–648 cm^−1^) were tested on the imaged areas of cell lines fixed in ethanol. Non-cell spectra were possible to be excluded based on mean transmission values being above 90%. Feasibility of a mean transmission based spectra filtering method with principal component analysis and linear discriminant analysis was shown to separate cell lines representing different tissue types. Fingerprint region resulted the best separation of cell lines spectra with accuracy of 99.84% at 70–75 mean transmittance range. Our approach in vitro was able to separate unique cell lines representing different tissues of origin. Proper data handling and spectra processing are key steps to achieve the adaptation of this dye-free technique for intraoperative surgery. Further studies are urgently needed to test this novel, marker-free approach.

## Introduction

Malignancies represent a huge burden on the society and the costs of novel oncological therapies are ever increasing [1]. Moreover, precise, personalized and fast multidisciplinary team decision is required to start the proper therapy [2].

Positive surgical margin (PSM) means that the resection margin is not tumor-free. This information is delivered by the pathology report and influences the therapeutical decision [3]. In the operating rooms the routine pathology background is not available on site, however, there is an urgent intraoperative clinical need to gain information on resected tissues, especially regarding the margins. In such cases intraoperative questions are raised to be answered by the pathologist who is situated in another location or building to where fresh tissue samples must be presented for histopathology. Frozen sections are cut and stained in the pathology department, which procedure takes about 15–30 min while the surgeons are waiting for answers in order to reach clinical decision. This procedure lengthens the operation time and the frozen sections deliver less precise morphology due to the lack of proper dehydration, which increases the uncertainty of diagnoses.

There are intriguing directions concerning the development of imaging techniques that might be integrated into routine pathology. These state of the art novel technologies include mass spectrometry, vibrational microscopy, multi-photon microscopy and different applications of confocal technologies. Several devices and their applications aiming at intraoperative imaging and identification of tissue types have reached the developmental stage, which allows the testing of the potential of these technologies in the operating rooms. Development of novel methods has recently entered not only into the phase of experimental setups but also that of possible application as diagnostic tools. Mass spectrometry analysis of molecules gained by evaporation of tissues is a destructive analytic method [4].

Infrared spectroscopy covers a spectral range over 780 nm. This spectrum is conventionally divided into near-, mid- and far infrared ranges (NIR 780–2500 nm, MIR 2500–25,000 nm, FIR higher than 25,000 nm, respectively). The lower the wavelength, the higher the energy of the light. In the past decade, tumorous specimens have been increasingly investigated by infrared spectroscopy based imaging [5–7]. The most frequently used application is still NIR, coming with more irradiated energy than MIR and thus deeper penetration in tissues [8]. Labelled and label-free approaches are used. There are known and biocompatible NIR active dye molecules available. Indocyanine green is the most frequently used in animal models [9] and its human application has been tested on liver carcinomas [10]. However, label free MIR is more promising for future practical application.

MIR spectroscopy fits more into the conventional pathological information, since the MIR photons contain less energy, consequently their spatial penetration is shorter, moreover, the signal-to-noise ratio of the MIR spectra is about two orders of magnitude higher than in case of NIR. In other words, the analysis of diagnostic MIR spectra is more reliable.

The mid-infrared area (4000–650 cm^−1^) includes the so-called fingerprint region (1800–400 cm^−1^) where peaks representative for lipids, protein, amide I/II and nucleic acids [11]. As the name suggests, fingerprint region is a specific part of the spectrum, generally containing most of the peaks.

Accordingly, the medical application of MIR is fewer than for NIR. MIR optical fibers have been commercially available since 2016, whereas previously laboratory tools existed only [12–14]. In a study, breast cancer imaging of 15 patients was carried out using mid- and long wave infrared cameras. The tumorous regions of the breast were identified using a Quantum Well Infrared Photodetector (QWIP) camera system [15]. In another study, urine samples from a small cohort of healthy women as well as female patients with gynaecological malignancies were investigated with MIR resulting in a high value of diagnostic accuracy using Principal Component Analysis (PCA) with support vector machine and genetic algorithm together with Linear Discriminant Analysis (LDA) algorithms [16]. The effect of basic tissue processing of pathological specimens (drying, formalin fixation, ethanol dehydration) on MIR spectrum was tested and an imaging protocol was proposed by Zahdi et al. [17]. Further investigations highlighted the pitfalls and best practices of tissue preparation methods for FT-IR spectroscopic analysis [18].

Gaydou et al took mid infrared imaging as a tool to investigate cell lines according to their infrared signature [19]. The cells were fixed in formalin and embedded into paraffin after centrifugation. Slices 8 μm thick were cut and their infrared image acquired. From their samples, 2 spectral images were collected, one from the cell culture and another from the paraffinized one. For data pre-processing extended multiplicative signal correction (EMSC) was used to correct the spectra mathematically.

Mid-infrared imaging could be used to differentiate cell lines and could be a promising technique for in vivo image analysis of tumours in animal models. This would be a key step to achieve the final goal to make this dye-free technique applicable for intraoperative surgical procedures [20, 21]. This means, that resection margins can be assessed by MIR characteristics.

The purpose of the present study was to prove that characteristic fingerprint regions are able to separate different cell lines representing normal and tumorous tissues by using label-free mid-infrared imaging.

## Materials and Methods

### Aluminium Coated Slides

Thin-film metal layers were deposited onto glass slides by vacuum evaporation to gain mid-infrared reflective surface. An electron-beam evaporation source was applied in a high-vacuum chamber, in which the glass slides were fastened onto the rotary sample holder. Aluminium was evaporated at 10^−4^ Pa for 20 min at an accelerating voltage of 7 kV and beam current of 200 mA, resulting in a layer thickness of ca. 150 nm.

### Cell Cultures

A-375 melanoma (Suppl. Fig.1a) and HT-29 colorectal human cancer cells (Suppl. Fig.1b) were obtained from ATCC (Manassas, Virginia, USA). The CCD986SK fibroblasts (Suppl. Fig.1c) (human from ATCC) were selected as a non-neoplastic cell line for comparison. Cell media used were Dulbecco’s modification of Eagle medium DMEM (Lonza Group Ltd., Basel, Switzerland) supplemented with 10% fetal bovine serum (FBS) (Euroclone Ltd., Pero, Italy) and cells were kept in a humidified incubator at 37 °C and 5% CO_2_. For infrared imaging evaluation cells were trypsinized and cell suspension was dropped on UV sterilized aluminium coated slides. Slides were placed in Petri dishes and immersed in phosphate-buffered saline (PBS), Whatman paper was used to avoid evaporation and the cells were allowed to attach overnight in the incubator. On the following day, cells were fixed using ethanol (EtOH) then washed with PBS and allowed to dry.

**Fig. 1:**
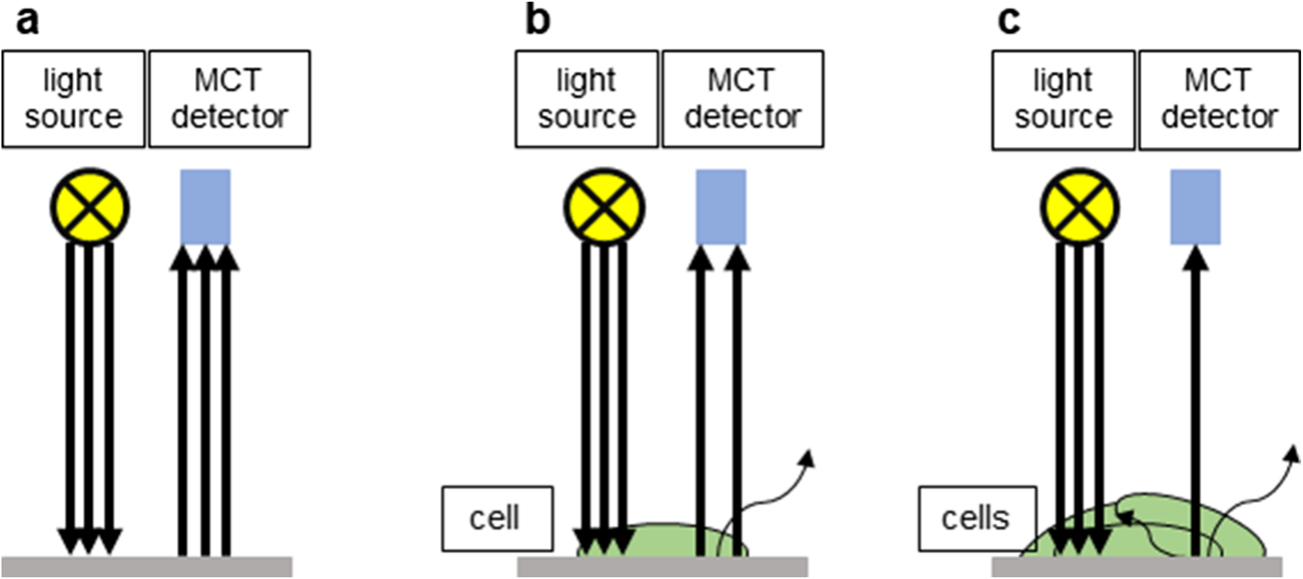
Intensity of the scattering light while travelling through more cells. **a**: principle of transflectance **b**: transflectance of single cell layer **c**: overlapping cells resulting in weak signal (low mean T%). Spectra of **a** and **c** were removed and final analyses were done on single cell layer **b**

### Infrared Imaging

The wavelength range of NIR is defined from 780 to 2500 nm (12820–4000 cm^−1^ – since due to the dispersed Fourier-transform (FT) spectrophotometers the wavenumber is typically measured in units of cm^−1^), the wavelengths of MIR are between 2500 nm and 25,000 nm (4000–400 cm^−1^) and the FIR range is between 25 and 1000 μm (400–10 cm^−1^). The higher the wavenumber, the higher the energy of the light. NIR and MIR photons elevate the chemical bonds to higher energy level, causing deformation motions (e.g. angular changes).

Fourier transform mid-infrared imaging was used for collecting spectra with transflection optical setup. Spotlight 400 microscope (Perkin Elmer Inc., Waltham, Massachusetts, USA) was connected to Spectrum 400 spectrophotometer used for scanning images. The Mercury Cadmium Tellurite (MCT) detector collected spectra with 4000–648 cm^−1^ wavelength range using step 4 cm^−1^, resolution 8 cm^−1^. The 300 μm × 550 μm images were scanned by pixel size 6.25 μm × 6.25 μm and 32 scans per pixel. A single image contained 48 × 88 pixels and resulted 4224 spectra. The same size areas were selected to image the three cell lines attached on three separate slides.

### Data Processing

#### Principal Component Analysis

Principal component analysis is an unsupervised variable-reduction technique. The different effects of the variables can be visualized through loadings and the scores represent the samples. A total of 12 PCA models were built to analyse our data. The obtained spectra can be collected into a table, where the rows can be referred to as samples and the columns as variables. In case of a whole range of measured IR spectra there are 838 wavenumbers (4000–648 cm^−1^) considered as variables. An infrared image contains a large amount of data: x and y set pixel location and each optical frequency band is described by a variable. This multidimensional dataset can be processed via data/dimension reduction to keep the variability. Every spectrum is represented by principal component scores and can be visualized by plots. Colouring the points makes human understanding easier. Unscrambler X 10.4 (CAMO Software AS, Oslo, Norway) software was applied to perform the PCAs and LDAs.

#### Atmospheric Correction and Noise Reduction

The presence of H_2_O and CO_2_ are mid-infrared absorbing molecules, hindering the identification of some analytes [22]. The acquired images were treated with two built-in algorithms of the SpectrumIMAGE R1.6.5.0396 software (Perkin Elmer Inc., Waltham, Massachusetts, USA) for atmospheric correction and noise reduction. The atmospheric CO_2_/H_2_O suppression by the least square fitting of the algorithm effected the atmospheric correction of the spectra [23]. The noise reduction was based on a 20-factor PCA. Since the noise has lower weights, the 20-factor based reconstructed spectrum is noise reduced. This method does not lead to the broadening of the spectrum peaks, unlike smoothing.

#### Linear Discriminant Analysis

Linear Discriminant Analysis is a supervised classification method. 12 LDA models were also created and run for further analysis of our data. LDA is the simplest of all possible classification methods that are based on Bayes’ formula [24]. It is based on the normal distribution assumption as well as on the assumption that the covariance matrices of the two (or more) groups are identical. The linear method is used when the difference between two groups can be represented by a linear function. The confusion matrix is a matrix used for visualization of classification results from supervised methods such as linear discriminant analysis classification. It carries information about the predicted and actual classifications of samples, with each row showing the instances in a predicted class, and each column representing the instances in an actual class. The projected spectra can be visualised using two-dimensional spaces. The points lying close to zero for a class are associated with the class.

#### Accuracy and Cohen’s Kappa Method

The performance of classification models are most often described by their confusion matrix – also known as error matrix. Sensitivity and specificity are interpretable with a 2 × 2 table (binary classifier). Our model has three classes of data, the three cell lines. Diagonal elements are correctly classified, the off-diagonal elements represent the number of misclassified spectra. To compare the performance of the models, Cohen’s Kappa and accuracy are the two indicators. The higher the value the better the model performance. The overall accuracy answers the question how often the classifier is correct? Cohen’s Kappa is a metric describing the relation between observed and expected accuracy. It presents the performance of the classification. Landis and Koch consider 0–0.20 as slight, 0.21–0.40 as fair, 0.41–0.60 as moderate, 0.61–0.80 as substantial, and 0.81–1 as almost perfect [25].

## Results

Our novel method used cancer cell line MIR spectra, which were pre-processed with atmospheric correction and noise reduction and were divided into 6 groups according to their mean transmittance (T%) value, calculated from the whole 4000–648 cm^−1^ range. These groups of spectra are observable in Suppl. Fig.2. First, the large amount of measured spectra had to be prefiltered to eliminate the spectra from cell free pixels showing too high mean T%, this was then followed by removing spectra from pixels containing too thick cell layers (i.e. overlapping cells) resulting in too low mean T% (Fig. 1). Therefore, the finally analysed population represented a systematically reduced number of spectra.

**Fig. 2:**
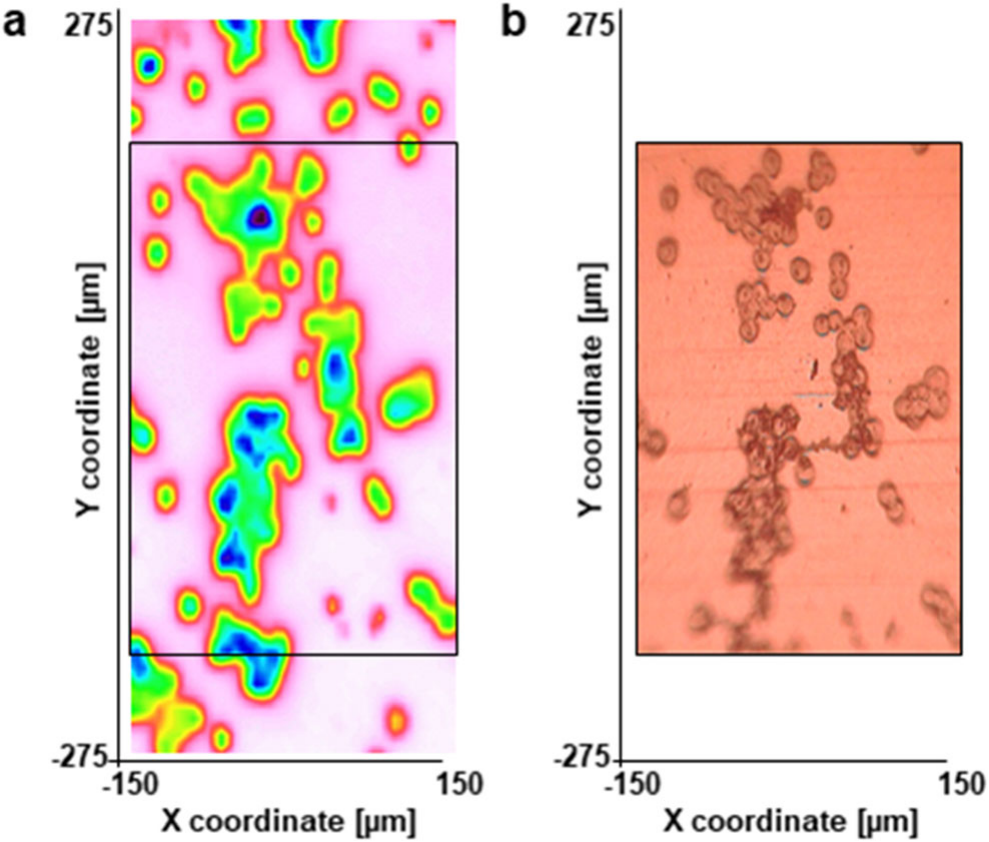
Representative images of ethanol fixed HT-29 cells. **a**: The complete measured area showing cell-free areas in pink, cells in green and the thick high absorbing regions as blue **b**: visible light microscopy image of the marked area on panel **a**

In case of HT-29 carcinoma cells some regions were cell-free (Fig. 2a, the pink areas), some were absorbing too much light (Fig. 2a, the blue areas).

The final analysis of acquired images was filtered by the mean transmittance. In case of A375 melanoma cells, the first group comprised the 0–50% mean T% range, which was followed by increasing 10% steps till 100% (Suppl. Fig.2). The spectra with lower than 50 T% were not useful because the peaks were deformed (e.g. ‘U’ shape of NH bond instead of typical ‘V’ shape at 3500–3300 cm^−1^ range). These same groups of the cell images will be packed together for further analyses. The remaining 50–80% range was divided into 6 pieces of 5 T% narrow groups (i.e. 50–55%, …, 75–80%). For each group, PCAs and LDAs were performed on all combinations of the spectral range. The reflecting cell-free surface showed high transmittance (>80%).

**Fig. 3:**
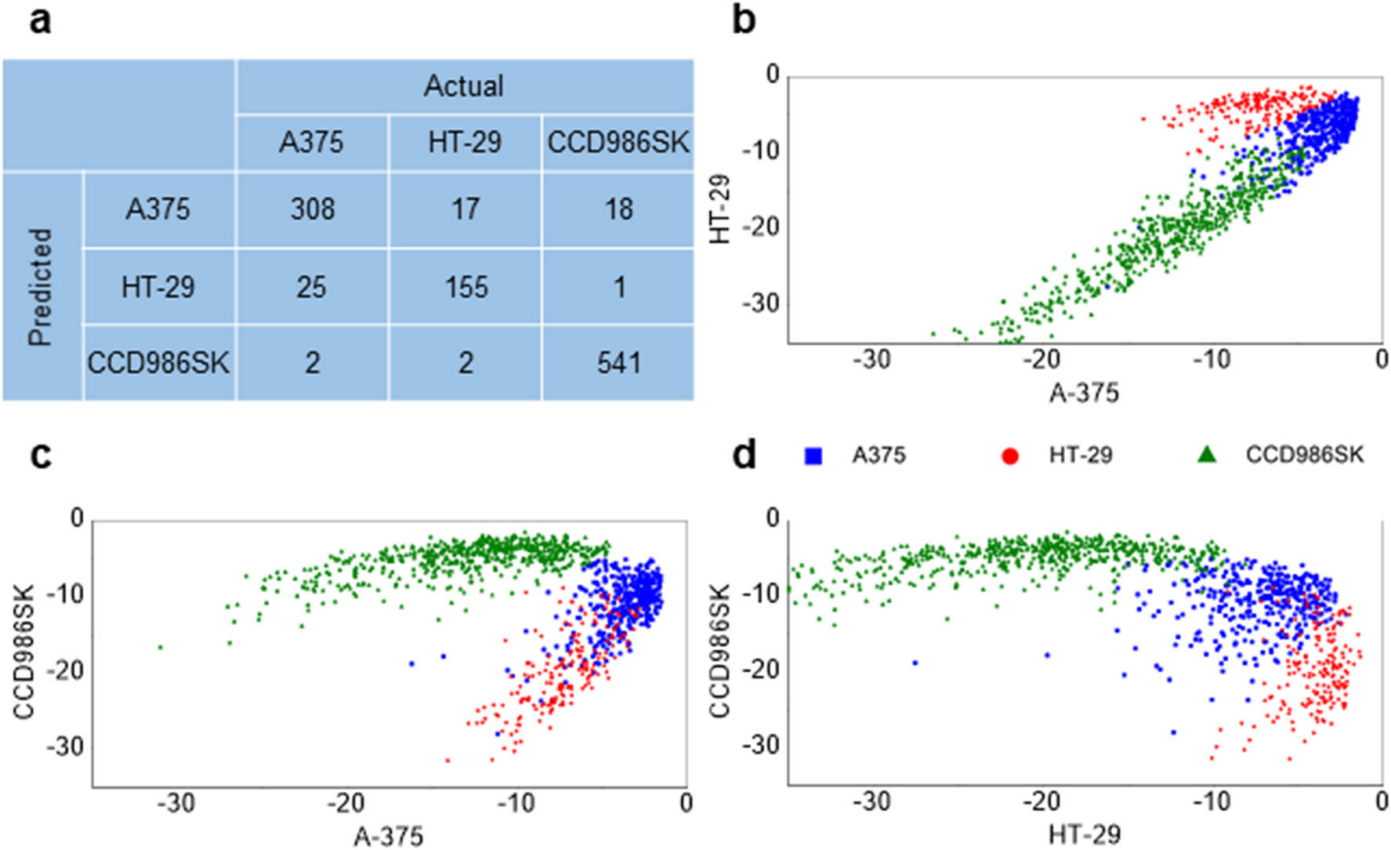
**a:** Confusion matrix of the whole region 65–70 T% LDA. **b**, **c** and **d** are the two dimension plots of projected scores coloured by the cell lines (A-375 – blue, HT-29 – red, CCD986SK – green)

Analysing the 5% ranges of human cancer and normal cell spectra with PCA the 50–55 T% range (out of 6 systematically tested 5% ranges of spectra) resulted in the best separation (Suppl, Fig. 3). The 1800–648 cm^−1^ region performed better than the whole spectrum range. The fingerprint region (1800–648 cm^−1^) absorption bands represented the biological complexity of samples in a more pronounced manner and allowed better separation. The purpose of PCA discrimination in our method is the visual testing of the separation, to present qualitative results. The number of misclassifications must be interpreted with the actual classes. For this reason, LDA is the chosen supervised method to classify the cells. Figure 3a shows the confusion matrix of the whole region 65–70 T% range results. Only 19 non-transformed cell representing data were misclassified. Figure 3b–d display the spectra points on two-dimension plots. The tumour representing groups mingled with each other, these moderated the accuracy, which was 93.92%. Fingerprint region analysis always resulted in higher accuracy then whole spectrum analysis. The supervised LDA models showed satisfying results on all mean transmittance datasets, both concerning accuracy and Cohen’s Kappa values, which were high. The exact values are listed in Table 1 and visualized on Suppl. Fig. 4. The best separation was reached in the fingerprint region with 70–75 T% range resulting 99.84% accuracy. All LDA model confusion matrices are shown in Suppl. Table 1.

**Table 1:** Accuracy and Cohen’s Kappa values of the built LDA models

	50_55 T%	55_60 T%	60_65 T%	65_70 T%	70_75 T%	75_80 T%
**LDA accuracy(%)**						
Whole region 4000-650 cm^−1^	89,220	89,160	92,150	93,920	92,650	91,260
Fingerprint region 1800-650 cm^−1^	99,400	99,510	97,970	99,250	99,840	99,790
**Cohen’s kappa**						
Whole region 4000-650 cm^−1^	0,827	0,825	0,872	0,899	0,880	0,858
Fingerprint region 1800-650 cm^−1^	0,990	0,992	0,967	0,988	0,997	0,997

## Discussion

Complete resection of solid tumours is usually associated with the best quality of life and possible long term survival of tumour patients. Precise evaluation of the tumour extent and surgical resection margins requires not only intraoperative macroscopical observation but histological verifications as well. Routinely, this is achieved by frozen section technology. There is a constant need for intraoperative on site method to assess the resection margins, however, so far no reliable method gained acceptance in the daily practices of surgical routine. Jansen-Winkeln et al demonstated a promising dye-free hyperspectral imaging feasibility study involving 20 patients who underwent colorectal surgery to determine the resection margin [26].

In our study, label-free mid infrared imaging was used to acquire spectra from three human cell lines after ethanol fixation: two different cancer cell lines and one fibroblast cell line as control. The informative spectra with good signal-to-noise ratio were selected according to the mean T% of the whole measured wavenumber region (4000–648 cm^−1^).

In the present study, we approached the problem from a general point of view that narrowed the whole image spectra into an informative subgroup. Mean T% based selection is not common, since mostly manual selection is used. The advantage of the applied T% selection is its objectivity and reproducibility. This basic mathematical approach allowed us to get rid of the aluminium background with small T% as well as of the deformed spectra, owing to e.g. overlapping cells, therefore, we were finally able to select the appropriate spectra of cells. The spectrum with the fingerprint region might be specific for different normal tissues as well as for different pathological alterations including benign and malignant tumours. This depends on the characteristics of the fingerprint region and might identify the tissue type or lesion.

The data analysis of Gaydou et al is in contrast with our approach since we filtered the spectra instead of correcting them [19]. Due to filtering, the number of spectra was reduced, however, the number of spectra remained unchanged during correction, while some wavelength values were modified by the EMSC algorithm. Quality input spectra for the analysis could be reached by either filtering or correction approaches. Our novel idea was to calculate the mean T% value from each spectrum and test this value during the filtering.

The limitation of our model was the transflectance imaging set-up using aluminium coated glass slides. A possible diagnostic tool with a built in endoscope could be based on attenuated total reflection [27] or simple transmission by encompassing the tissue.

PCAs were performed on all the investigated subgroups on both the whole (i.e. 4000–648 cm^−1^) and fingerprint (*1800*–648 cm^−1^) regions. As a visual result the 50–55 mean T% range, 1800–648 cm^−1^ spectra obtained from ethanol fixed cells presented the best grouping. We successfully demonstrated the feasibility of our infrared method to separate different human cell types by their filtered mid-infrared spectra with highlighted range. Non-linear supervised computation intensive models (e.g. artificial neural networks) have spread fast, however, it is not easy to interpret the results and effects of the variables. In addition, the risk of overfitting is much higher than by using PCA [28]. The first two factors described for the spectra points for the grouping were similar in the majority of our cases, with some overlaps observed in our cases too. In conclusion, our recommendation is to use the 1800–648 cm^−1^ MIR spectral range for diagnostic applications. Based on our results mean T% based selection should be considered as a spectrum pretreatment before analysis.

LDA was chosen as supervised classification method, which performed satisfactorily on all investigated mean transmittance ranges. The fingerprint region accuracy and Cohen’s kappa values were constantly higher than the whole region model results. These indicators of the LDA model have shown an excellent power of classification.

The tumour microenvironment and the normal cell-free matrix were not investigated, since only tumour cells and normal cells were compared to each other. As a next step, numerous different normal and transformed cell type spectra should be collected into a spectra library. The challenging issue is to identify and image the tumour-infiltrating immune cells in the stroma of the tumour [29].

Our novel approach showed that after using a generalized spectra filtering the proper supervised or unsupervised mathematical analysis enables the separation of normal and tumorous samples even in case of partially overlapping spectra. This novel, marker-free approach and the fiber development will facilitate the spread of new diagnostic in vivo applications of mid-infrared imaging tools. Our in vitro data suggests that this novel method can be further developed into an in vivo testing system which requires further investigations.
